# A network dynamical simulation model for the study of antibiotic resistance in microbial communities

**DOI:** 10.1080/19490976.2026.2734703

**Published:** 2026-09-19

**Authors:** Miguel Atl Silva-Magaña, Lorena Patricia Mora-Flores, Marco A. Pita-Galeana, Enrique Hernández-Lemus, Guillermo de Anda-Jáuregui

**Affiliations:** a Computational Genomics Division, National Institute of Genomic Medicine, Mexico City, Mexico; b Researchers for Mexico Program, SECIHTI, Mexico City, Mexico

**Keywords:** Enterobacter interaction network, microbiome networks, antimicrobial resistance, antibiotic perturbation, microbial community dynamics

## Abstract

Antibiotic resistance emerges from ecological and evolutionary processes occurring within complex microbial communities. Interactions among microorganisms can shape the pathways through which resistance traits spread and persist, yet many theoretical approaches treat microbial populations as homogeneous compartments. Here we present a simulation-based network dynamical model that represents microbial communities as ecological association networks. In this formulation, nodes correspond to bacterial populations, metapopulations, or taxon-level ecological units, while resistant counterparts represent state-expanded subpopulations associated with the same ecological unit. Edges represent co-occurrence-based ecological proximity rather than direct physical contacts or confirmed horizontal gene transfer events. Using stochastic simulations across multiple network topologies, we explore how structural properties of microbial communities influence the emergence and persistence of resistance. Parameter sweeps across transmission probability, initial resistance fraction, and antibiotic intervention timing allow us to characterize regimes in which resistance either remains localized or spreads through the community. The model produces time series of resistant and susceptible states and snapshots of evolving network configurations, enabling qualitative comparison across simulation scenarios. Our results show that network structure strongly modulates resistance dynamics. Highly clustered networks tend to trap resistance within local neighborhoods, whereas heterogeneous networks with hub nodes facilitate rapid dissemination. Antibiotic perturbations can either suppress resistance or paradoxically accelerate its expansion depending on network topology and intervention timing. These findings should be interpreted as qualitative results from a minimal proof-of-concept model, not as a direct reconstruction of plasmid transfer, species replacement, or patient-specific microbiome responses.

## Introduction

Antibiotic resistance is widely recognized as one of the most pressing challenges in modern medicine.^1-3^ The consequences of resistance extend well beyond the clinical management of individual infections: they reconfigure the very ecological landscape in which pathogens evolve, alter the selective pressures acting on entire microbial assemblages, and ultimately threaten to undermine the therapeutic foundations upon which contemporary medicine rests.^4-6^ Projections estimating millions of deaths attributable annually to resistant infections are not merely alarming statistics, as they reflect the tangible outcome of evolutionary processes that have been inadvertently accelerated by decades of antibiotic misuse, agricultural overexposure, and incomplete treatment courses.^7^ ^,^ ^8^ Understanding the mechanisms that govern the emergence and propagation of resistance is therefore not only a matter of basic scientific inquiry but one of immediate and urgent public health relevance.^9^ ^,^ ^10^

While resistance is often studied at the level of individual pathogens, microorganisms typically exist within complex ecological communities such as the human microbiome.^11-13^ This distinction is far from trivial. The microbial community is not simply a backdrop against which individual organisms evolve in isolation; it is an active ecological matrix that shapes evolutionary trajectories, modulates selective pressures, and provides both physical and biochemical contexts within which resistance traits are gained, lost, transferred, or suppressed.^14^ ^,^ ^15^ The human gut microbiome, for instance, harbors extraordinary phylogenetic diversity packed into a densely connected system where spatial proximity, metabolic dependencies, and competitive exclusion interact simultaneously across multiple scales.^16-19^ Within these communities, microbial taxa interact through metabolic exchange, ecological competition, and shared environmental niches.^11^ ^,^ ^13^ ^,^ ^20^ These interactions create structured systems in which evolutionary and ecological processes unfold in ways that simply cannot be anticipated from single-species analyzes.

Among the mechanisms by which resistance spreads within such communities, horizontal gene transfer occupies a position of particular importance.^21^ ^,^ ^22^ Conjugative plasmids, transposons, integrons, and other mobile genetic elements permit the lateral movement of resistance determinants across phylogenetic boundaries that would otherwise constrain vertical inheritance.^23-25^ This means that a resistance gene confined to a rare or ecologically marginal taxon can, under the right conditions, disseminate rapidly to numerically dominant or ecologically central members of the community, bypassing the ordinary requirements of clonal expansion.^26-28^ However, the routes followed by resistance genes are not determined by network topology alone; they also depend on phylogenetic relatedness, donor-recipient compatibility, and the transmissibility of mobile genetic elements.^27^ ^,^ ^29^ Thus, microbial network structure should be viewed as an ecological scaffold that can shape opportunities for resistance propagation, not as a direct map of gene-transfer routes.^30^ Recognizing this connection between community structure and evolutionary dynamics motivates the development of explicitly network-based models for studying resistance.

Recent advances in sequencing technologies have made it possible to reconstruct microbial association networks from co-occurrence patterns observed across samples.^12^ The inference of microbial ecological networks from metagenomic and 16S rRNA amplicon datasets has become a mature methodological enterprise, yielding association graphs in which pairwise relationships between taxa reflect statistical dependencies and ecological proximity. Such networks can provide a useful representation of community structure, but their edges should not be interpreted as direct physical contacts, confirmed conjugative pairs, or validated horizontal gene transfer routes.^31-33^

Measures of centrality, clustering, modularity, and degree distribution have all been brought to bear on these data, revealing that microbial communities exhibit non-random topological features, for instance, hubs of high connectivity, densely woven modules corresponding to co-adapted consortia, and long-range connective pathways that bridge otherwise isolated sub-communities. However, translating these static representations into dynamical models capable of exploring ecological perturbations remains an open challenge. Network reconstruction from cross-sectional data captures a snapshot of community structure, but the ecological consequences of that structure under perturbation, antibiotic treatment being a paradigmatic example, depend on dynamics that static analyzes alone cannot reveal.

In parallel, theoretical work in network epidemiology has shown that the spread of traits or pathogens can be strongly influenced by the topology of the underlying contact network.^34-36^ Beginning with the seminal work of Pastor-Satorras and Vespignani^37^ on scale-free networks and extending through decades of developments in complex systems theory,^38^ it has become clear that the heterogeneity of node degree distributions profoundly affects epidemic thresholds and the conditions under which outbreaks are self-sustaining. Concepts such as clustering, degree heterogeneity, and average path length determine how quickly a contagion spreads and which nodes become critical for transmission.^39^

Highly clustered networks tend to confine spreading processes within tightly connected neighborhoods, effectively creating reservoirs that are insulated from global perturbations. Heterogeneous networks featuring hubs, nodes with disproportionately many connections, conversely enable rapid long-range dissemination, lowering effective thresholds for system-wide invasion. These ideas provide a natural framework for studying resistance-associated states within microbial communities. Although microbial gene transfer is biologically more complex than simple contagion, the mathematics of stochastic transmission on graphs provides a useful abstraction for comparing resistance propagation across network topologies.^40^

A further complication, and one that distinguishes the microbial resistance problem from classical epidemic models, is the dual action of antibiotic perturbations. Antibiotics do not function purely as selective filters that eliminate susceptible organisms while leaving resistant ones untouched.^41^ They alter the composition of the entire community, disrupting ecological balances that may otherwise constrain the proliferation of resistant strains, releasing nutrients and physical niches previously occupied by susceptible competitors, and potentially inducing stress responses that upregulate horizontal gene transfer. The net effect of antibiotic exposure on community-level resistance dynamics is therefore not determined solely by the pharmacological properties of the drug and the susceptibility of individual strains, but by the ecological consequences of community disruption,^14^ i.e., there are consequences that depend in a non-trivial way on the structure of microbial interactions. Capturing this interplay between pharmacological perturbation and network-mediated ecological dynamics requires models that integrate both dimensions explicitly.

Previous theoretical approaches to antibiotic resistance have tended to work within one of two traditions: population genetics models that track allele frequencies under selection and mutation, and pharmacodynamic models that couple bacterial killing rates to drug concentrations.^42^ ^,^ ^43^ Both traditions have yielded important insights, but both also treat the microbial population as a more or less homogeneous compartment, abstracting away the structural heterogeneity of community associations that, as argued above, can be decisive for resistance outcomes. Agent-based and individual-based models offer richer representations of spatial and social structure but carry steep computational costs and often resist analytical treatment.^44^ The network dynamical approach proposed here occupies a productive middle ground: it is sufficiently abstract to permit systematic parameter exploration and qualitative analysis, yet sufficiently structured to capture the topological features of real microbial communities.

In this study, thus, we propose a simulation-based network dynamical model to investigate how resistance propagates in microbial association networks under antibiotic perturbation. The model represents bacterial populations, metapopulations, or taxon-level ecological units as nodes and simulates stochastic processes that govern local resistance acquisition and antibiotic-induced perturbation. By systematically varying network topology and dynamical parameters, we analyze the conditions under which resistance spreads, persists, or collapses following treatment. The model is intended as a minimal proof of concept and does not aim to reproduce all molecular details of horizontal gene transfer, plasmid compatibility, or species-specific population dynamics. In doing so, we aim to bridge the conceptual gap between the ecological structure of microbial communities and the dynamical theory of resistance evolution, contributing to a more ecologically grounded understanding of one of the defining biomedical problems of our time.

## Methods

### Mathematical formulation of the model

#### Network definition

Let $G_{t}\;=\;(V_{t},\;E_{t})$ denote the ecological network at discrete time *t*, where $V_{t}$ is the set of nodes and $E_{t}$ the set of undirected edges.^45^ ^,^ ^46^ Each node represents a bacterial population or metapopulation, and each edge represents an ecological interaction between two populations.

In this formulation, a node should not be interpreted as a newly created bacterial species, strain, or taxonomic unit. Rather, it represents a bacterial population, metapopulation, or taxon-level ecological unit of unknown but fixed size within a given network context. This distinction is important because the model follows susceptible and resistant subpopulations associated with the same microbial unit, not formal speciation events.

Each node $i\in V_{\mathrm{ti}}$ has an associated resistance state $X_{i}(t)\in \{0,1\}$ where $X_{i}(t)=0$ if node *i* is susceptible to antibiotic AB, and $X_{i}(t)=1$ if node *i* is resistant to antibiotic AB.

At the initial time, the network is assumed to be in ecological equilibrium under stable environmental conditions, with no external stressors and constant population sizes.^47^ Thus, changes in the system arise only from resistance transmission and antibiotic perturbation, not from intrinsic population growth or decline.

The equilibrium assumption applies only to the initial ecological scaffold before resistance dynamics and antibiotic perturbation are introduced. Once the simulation starts, the system is intentionally driven away from this initial condition by resistance acquisition and antibiotic selection.

#### Resistance transmission

Let $N_{i}(t)$ denote the neighborhood of node *i* at time *t*,

$$\mathrm{Ni}(t)=\{j\;\in \;V_{t}:(i,j)\in E_{t}\}$$

At each time step, every resistant node *i* may generate a horizontal resistance transmission event according to a Poisson process with rate parameter *p*. If the number of transmission events generated by node *i* during one time step is denoted by

Yi(t)∼Poisson(λ=p)

then the probability that node *i* produces at least one transmission event at time *t* is

$$P(Y_{i}(t)\;\geq \;1)\;=\;1\;-\;e^{-p}$$

Whenever $Y_{i}(t)\geq 1Y_{i}(t)$ , one neighboring node $j\in N_{i}(t)\;$ is selected as recipient. In the simplest version of the model, the recipient is chosen uniformly at random from the neighborhood of *i*.

Then, resistance acquisition is modeled as a duplication event. A new node *j⁺* is added to the graph such that $X_{j^{+}}(t+1)=1.$ This new node inherits the ecological interactions of the original susceptible node *j*. Formally, if

$N_{j}(t)=\{k\in \mathrm{Vt}:(j,k)\in \mathrm{Et}\}$ , then the duplicated resistant node satisfies $N_{j+}(t+1)=N_{j}(t)$ .

**Figure 1.**
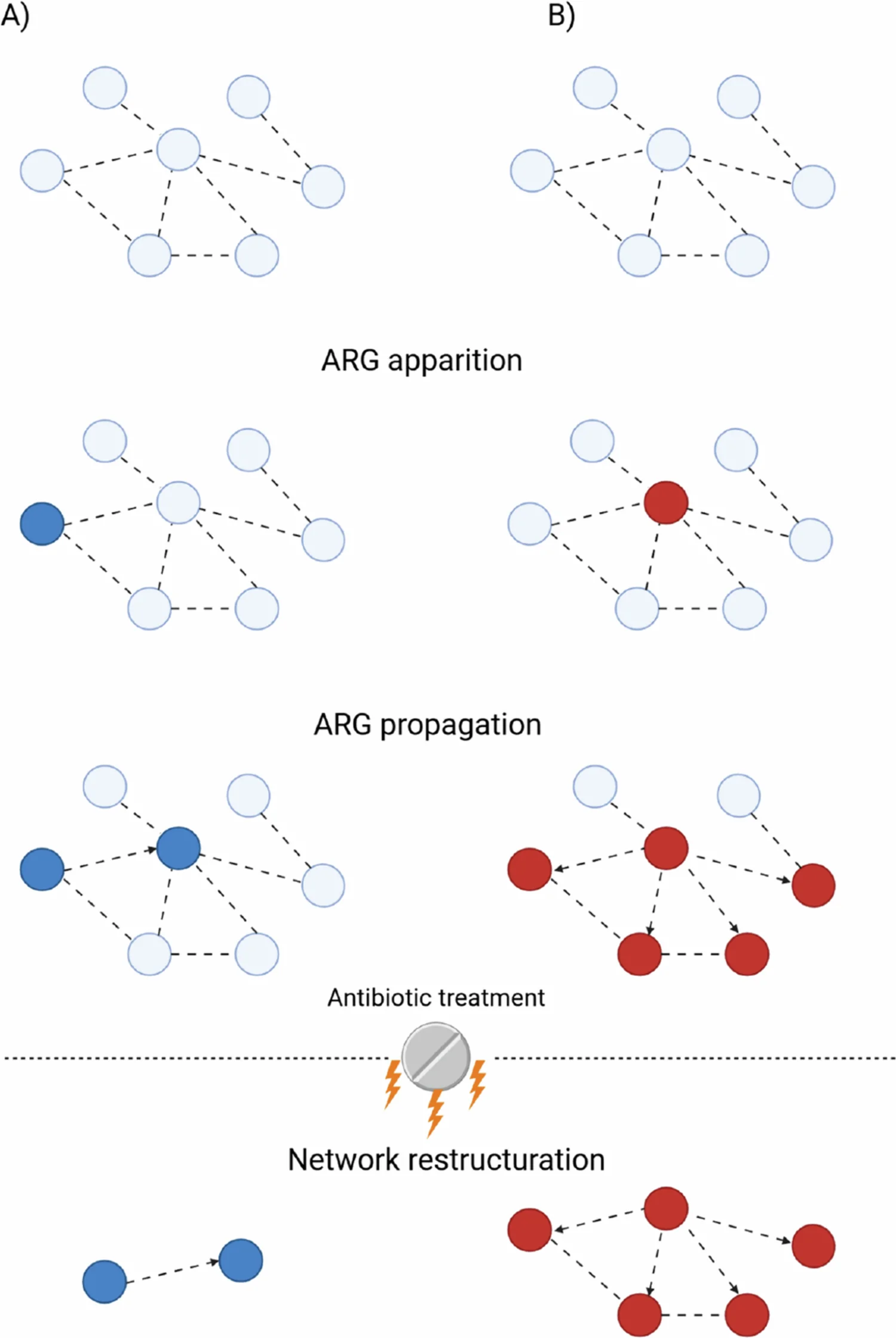
Modeled propagation of ARGs in a bacterial community. Conceptual representation of ARG dissemination within a microbial community depending on the connectivity of the initial carrier. A) A poorly connected peripheral bacterium limits ARG dissemination while B) A highly connected bacterial hub rapidly spreads AMR across the microbial network.

The original node *j* remains in the network and remains susceptible. Thus, in this implementation, resistance acquisition does not replace the susceptible population but creates a resistant counterpart coexisting in the same ecological neighborhood. If a susceptible population A gives rise to A′, A and A′ should be interpreted as susceptible and resistant subpopulations associated with the same bacterial type, not as two different species. Because A′ is assumed to arise within the same ecological context, it inherits the local associations of A. Therefore, resistance spread expands the modeled population-state space rather than the taxonomic composition of the community (see Figure 1).

#### Antibiotic perturbation

At a prescribed time *T*_*x*_ an antibiotic *AB* is administered. In the simplest case, the antibiotic is assumed to act instantaneously and to be perfectly effective against susceptible nodes while being completely ineffective against resistant nodes.

Thus, immediately after treatment, all susceptible nodes are removed, and resistant nodes are kept.

The post-treatment edge set is the induced subgraph on the surviving resistant nodes,

$$E_{T{⁺}_{x\;}}=\;\{(i,j)\;\in \;E_{T_{x}}\;:\;i\;\in \;V_{{T^{+}}_{x}},j\;\in \;V_{{T^{+}}_{x}}\;\}\;$$

This operation is equivalent to a site percolation process in which all susceptible nodes are deleted simultaneously from the network.

The time variable *t* denotes a discrete simulation update step. It should not be interpreted as a fixed number of hours, days, or generations unless the model is calibrated with longitudinal empirical data. In the present proof-of-concept analysis, t is used to compare relative dynamics across network structures and parameter regimes.

### Dynamical simulations across synthetic complex network models

To investigate how network topology influences the propagation of antibiotic resistance, we conducted dynamical simulations on synthetic complex networks generated using three classical models: Erdős-Rényi (ER) random graphs, Watts-Strogatz (WS) “small-world” networks, and Barabási-Albert (BA) scale-free networks.^39^

For each network family, graphs were generated by exploring a range of structural parameters. These included the number of nodes in the network and the parameters governing edge formation for each model. In the case of ER networks, graphs were generated using different values of the link probability *p (p_edge).* For BA networks, the parameter m, representing the number of edges added by each newly introduced node, was varied. For WS networks, simulations explored combinations of initial neighborhood size k and rewiring probability 𝛃.

Across these synthetic networks, we simulated resistance dynamics using the model described above while systematically varying key dynamical parameters. These included the probability of resistance transmission *p*, the initial fraction of resistant nodes k_0_, and the time of antibiotic administration *T*_*x*_.

The synthetic-network values of *p* were selected as exploratory low- and high-transmission regimes, rather than calibrated biological rates. They were used to test whether topology changes the qualitative behavior of the model under weak and strong local acquisition. For the empirical network, a broader range of *p* values was explored to visualize more gradual changes. Future work should use denser parameter sweeps to identify possible critical thresholds more precisely.

For each combination of network structure and dynamical parameters, multiple independent simulations were performed in order to capture the stochastic variability inherent to the process.^48^

During each simulation we recorded the temporal evolution of the system by tracking the number of resistant nodes through time, producing time series that describe the spread of resistance across the network. In addition, we characterized the structural impact of antibiotic treatment by comparing network topology before antibiotic administration and after the removal of susceptible nodes. Specifically, we quantified changes in global network properties such as:

– Edge density: proportion of realized edges relative to the maximum possible number of edges in the network.
– Clustering coefficient: tendency of nodes to form locally interconnected groups, quantified as the fraction of closed triplets (triangles) relative to all connected triplets in the network.
– Connectivity: overall fragmentation of the network, quantified by the number of connected components.
– Largest component size: number (or fraction) of nodes belonging to the largest connected component of the network.

### Dynamical simulations on an empirical microbial association network

To evaluate the behavior of the model in a biologically grounded setting, we performed dynamical simulations on an empirical microbial association network derived from human gut microbiome data. The network corresponds to the ecological structure underlying enterotype organization described by MetaHIT Consortium (additional members) et al.^49^ Microbiome data were accessed and processed using the {phyloseq} R package framework for microbiome analysis.^50^

From these data we obtained an ecological association network in which nodes represent microbial taxa and edges represent ecological associations inferred from co-occurrence patterns within the gut microbiome. The resulting graph was simplified to an undirected network and used as the fixed association structure on which resistance dynamics were simulated.

The empirical network was used as an illustrative ecological association network. The Jaccard distance threshold of 0.3 was selected to retain associations strong enough to define ecological proximity while preserving sufficient connectivity for dynamical exploration. Thus, the network should be interpreted as a statistical scaffold for simulating a coarse resistance acquisition process, not as a direct map of horizontal gene transfer contacts. Sensitivity analyzes using alternative thresholds would be valuable in future work.

We applied the same resistance dynamics model described above and explored a parameter space defined by the probability of resistance transmission *p* and the initial fraction of resistant nodes *k*0. For each parameter combination, multiple stochastic simulations were performed in order to capture the variability of the resistance propagation process.

In addition to varying these dynamical parameters, we investigated different strategies for selecting the initial set of resistant nodes. Three seeding regimes were considered:

• Random seeding: resistant nodes were selected uniformly at random from the network.
• Hub seeding: resistance was initialized preferentially in the highest-degree nodes, representing taxa with the largest number of ecological interactions.
• Spatially dispersed seeding: resistant nodes were chosen such that they were not immediate neighbors of one another, ensuring that the initial resistant populations were maximally separated across the network.

These seeding strategies allow us to examine how the topological placement of resistance influences the subsequent spread of resistance through ecological interactions.

As in the synthetic network experiments, simulations generated time series describing the number of resistant nodes through time. In addition, we quantified the structural impact of antibiotic treatment by comparing the topology of the empirical network before antibiotic administration and after the removal of susceptible nodes, thereby assessing how antibiotic perturbations reshape the ecological structure of microbial communities.

This empirical analysis provides a complementary perspective to the synthetic network experiments, allowing us to evaluate whether the qualitative behaviors observed across canonical network models also emerge in realistic microbial ecological networks.

### Workflow

**Figure 2.**
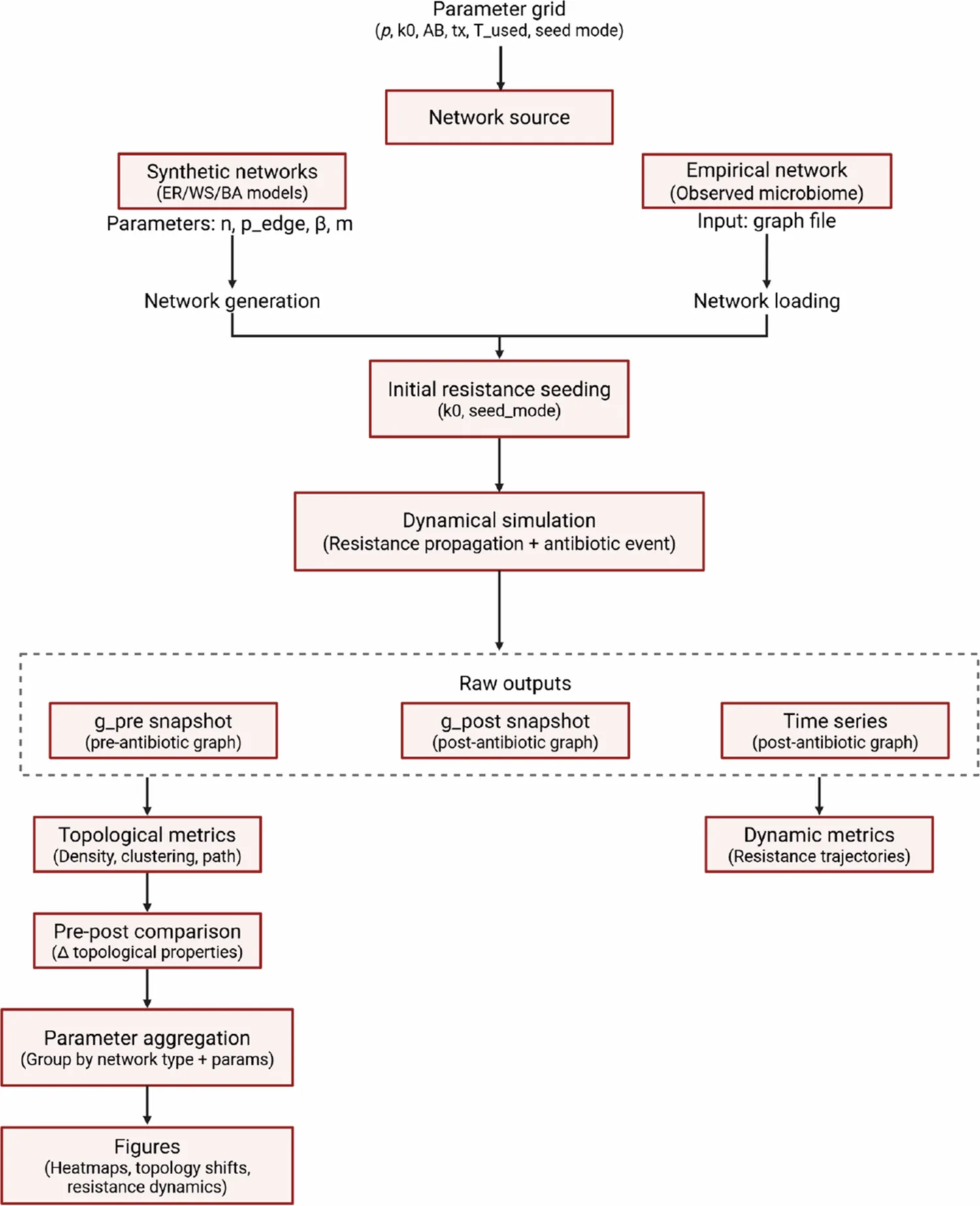
Methodological workflow for the simulation of antibiotic resistance spread dynamics.

In short, we implemented a computational workflow to perform the aforementioned simulations, which we summarize in Figure 2.

A methodological note on the computational implementation of these simulations may be found in Addendum A.

## Results

### Resistance spread is jointly determined by network topology and initial resistance conditions

**Figure 3.**
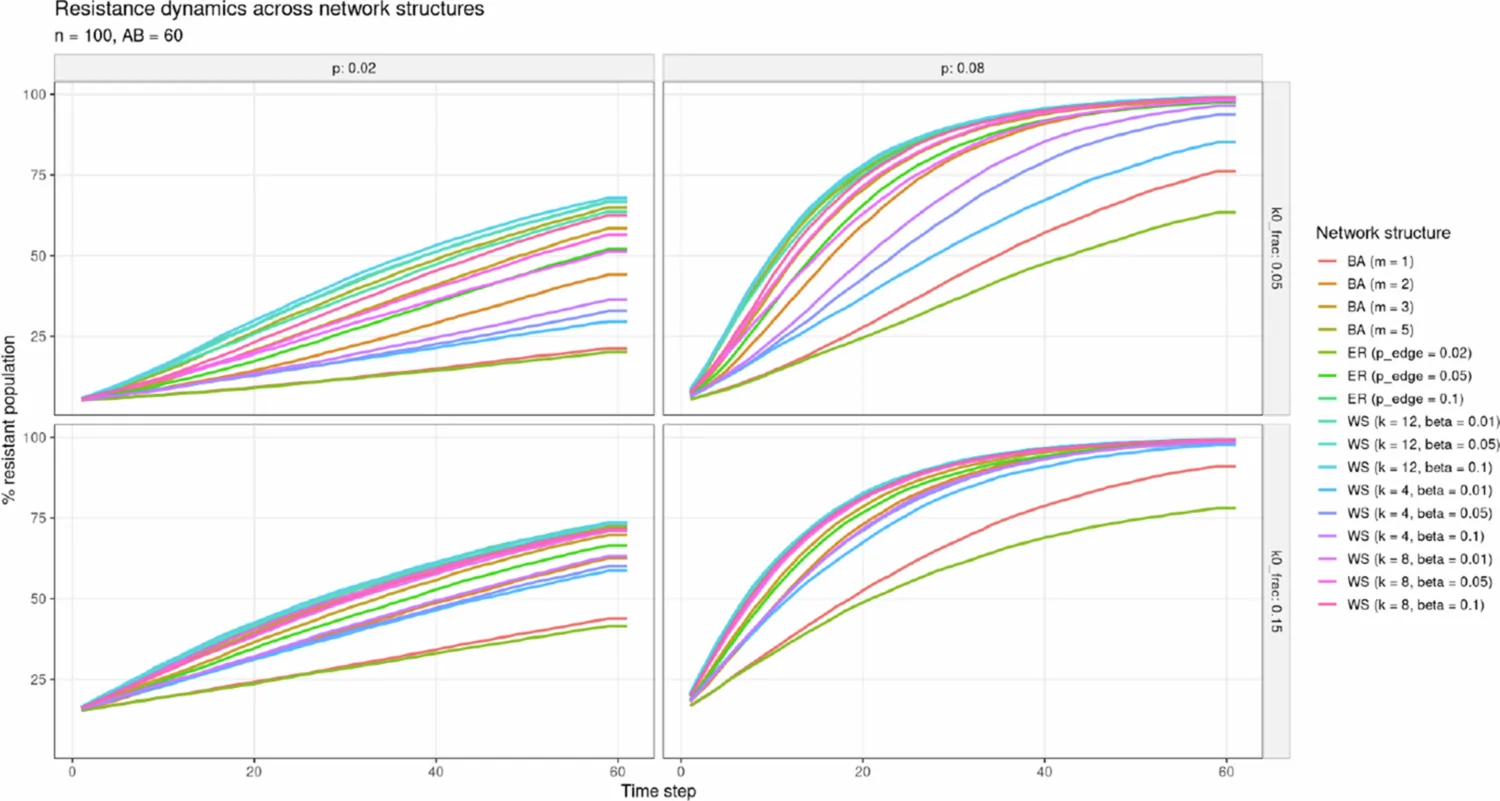
Time series of antibiotic resistant fraction. Each line represents the dynamics of resistance acquisition, measured as the fraction of nodes in the network that are resistant at any given time step. Lines are separated based on the initial network structure and associated parameters. Figure is split in panels based on the values of resistance transmission probability *p* and initial fraction of resistant nodes K_0_.

We first examined how resistance propagates across different network structures by simulating the model on synthetic graphs generated under Erdős-Rényi (ER), Watts-Strogatz (WS), and Barabási-Albert (BA) topologies. Figure 3 shows the temporal evolution of the fraction of resistant nodes across simulations under different combinations of transmission probability *p* and initial resistant fraction k_0_.

Across all network models, resistance spreads through the network as a cumulative process in which the fraction of resistant populations increases over time. However, both the rate of propagation and the final fraction of resistant nodes reached before antibiotic administration depend strongly on the interaction between network topology and the dynamical parameters governing resistance transmission.

The probability of transmission *p* exerts a strong control over the speed of the process. When transmission probability is low (*p* = 0.02), resistance spreads gradually and remains far from saturating the network within the simulated time window. In this regime, the resistant fraction grows approximately linearly across time and remains well below full network coverage. In contrast, when transmission probability is higher (*p* = 0.08), resistance spreads rapidly and approaches near-complete saturation of the network before the antibiotic intervention at t = 60. The resulting trajectories show a sigmoidal shape characteristic of contagion processes on networks.

The initial fraction of resistant nodes k0 also modulates the dynamics of spread. Increasing it accelerates the early phase of the process by increasing the number of initial sources of transmission. As a consequence, trajectories starting from larger initial resistant fractions reach high levels of resistance earlier and exhibit steeper growth curves. This effect is particularly visible in the high-transmission regime, where even modest differences in initial resistant fraction substantially alter the time required for resistance to spread through the network.

Beyond these dynamical parameters, the underlying network topology influences how efficiently resistance propagates. Networks with stronger local clustering or shorter average path lengths (such as small worlds) generally facilitate faster spread, as resistant nodes can reach larger portions of the network through fewer steps. Conversely, sparser or less interconnected networks exhibit slower propagation, resulting in lower resistant fractions at the time of antibiotic administration.

Taken together, these results indicate that resistance spread in microbial ecological networks is governed by the combined influence of transmission dynamics, initial conditions, and network structure. Even under identical dynamical parameters, differences in network topology can produce markedly different propagation trajectories, highlighting the importance of ecological association structure in shaping the emergence and expansion of antibiotic resistance within microbial communities.

### Antibiotic perturbation leads to network reconfiguration

Antibiotic administration induces a structural perturbation of the ecological association network by removing all susceptible populations simultaneously, leaving only resistant nodes and the interactions among them. In the model, this operation corresponds to a large-scale site percolation process, where nodes lacking resistance are deleted from the graph at the time of treatment. Because resistance spreads dynamically before the antibiotic is applied, the topology of the surviving network depends on how extensively resistance has propagated across the system prior to the intervention.

**Figure 4.**
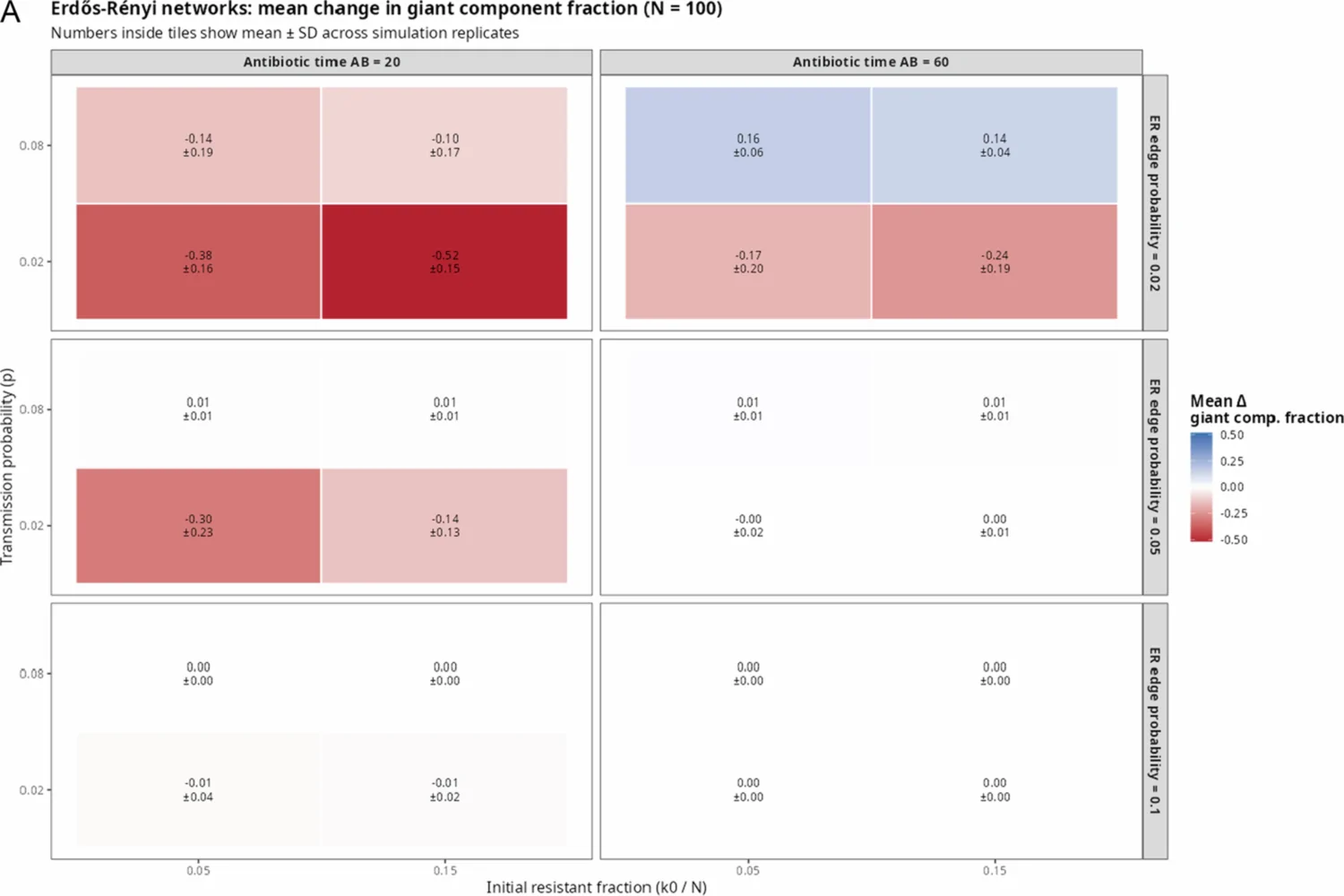
**A**: Heatmap showing the mean change in giant component fraction induced by antibiotic perturbation across simulation replicates (*N* = 100) in Erdős-Rényi networks. Within each panel, rows correspond to transmission probability (*p*) and columns correspond to the initial resistant fraction (*K*_*0*_ */N*), grouped by antibiotic administration time (*AB* = 20 or 60), and right-side labels indicate the ER edge probability that is the probability that any pair of nodes is connected when the random network is generated. Values are shown as mean ± SD. **B:** Heatmap showing the mean change in giant component fraction induced by antibiotic perturbation across simulation replicates (*N* = 100) in Watts-Strogatz networks. Within each panel, rows correspond to transmission probability (*p*) and columns correspond to the initial resistant fraction (*K*_*0*_ */N*). Panels are grouped by antibiotic administration time (*AB* = 20 or 60), and the right-side labels indicate the network settings used in each block (A–I), where *K* is the number of nearest neighbors initially connected to each node and *β* is the rewiring probability. Values are shown as mean ± SD. **C:** Heatmap showing the mean change in giant component fraction induced by antibiotic perturbation across simulation replicates (*N* = 100) in Barabási-Albert networks. Within each panel, rows correspond to transmission probability (*p*) and columns correspond to the initial resistant fraction (K_0_/N). Panels are grouped by antibiotic administration time (AB = 20 or 60), and the right-side labels indicate the attachment parameter m, defined as the number of links added by each new node when joining the network. Values are shown as mean ± SD.

**Figure 5.**
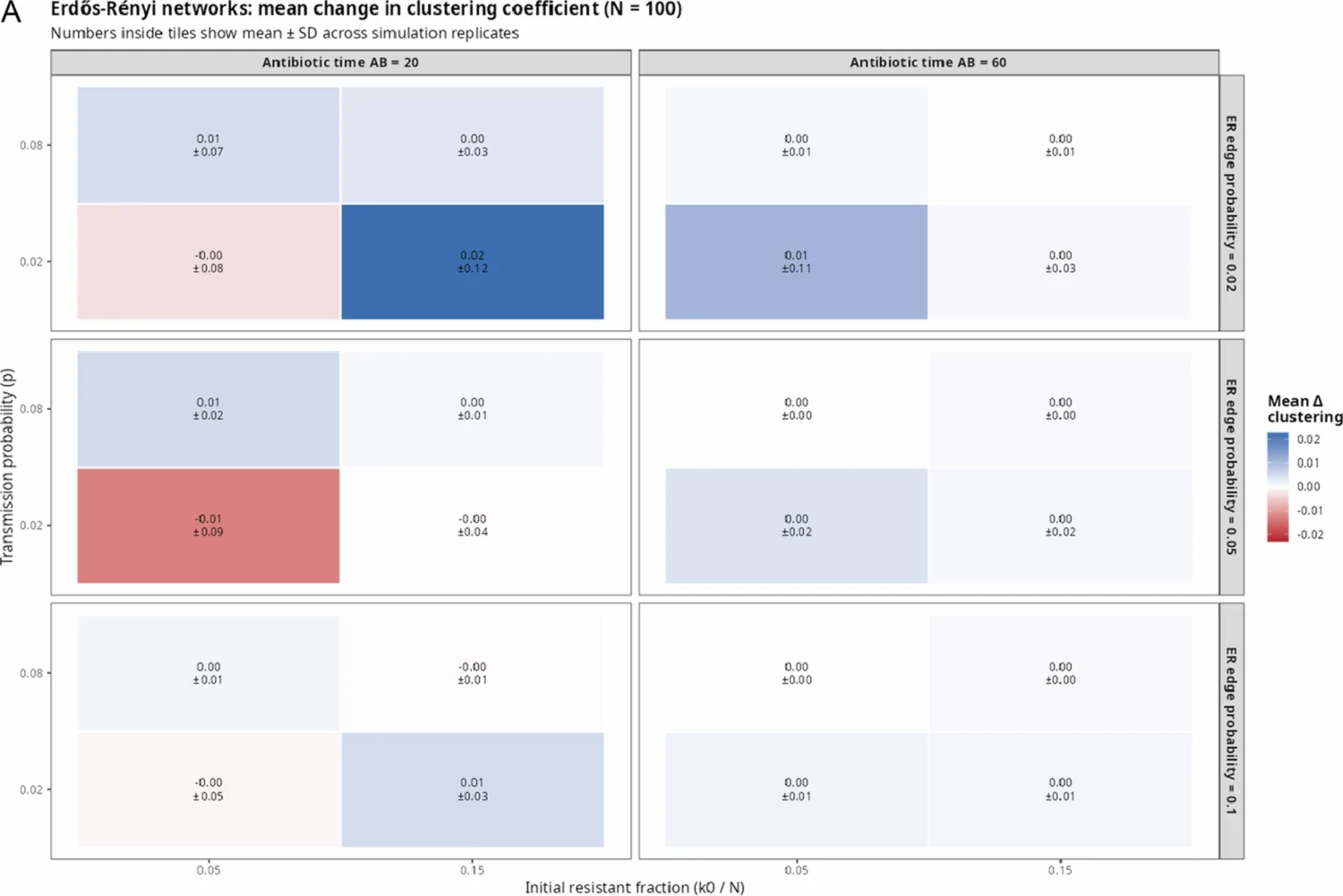
**A:** Heatmap showing the mean change in clustering coefficient induced by antibiotic perturbation across simulation replicates (*N* = 100) in Erdős-Rényi networks. Within each panel, rows correspond to transmission probability (*p*) and columns correspond to the initial resistant fraction (K_0_/N). Panels are grouped by antibiotic administration time (AB = 20 or 60), and the right-side labels indicate the ER edge probability, that is, the probability that any pair of nodes is connected when the random network is generated. Values are shown as mean ± SD. **B**: Heatmap showing the mean change in clustering coefficient induced by antibiotic perturbation across simulation replicates (*N* = 100) in Watts-Strogatz networks. Within each panel, rows correspond to transmission probability (*p*) and columns correspond to the initial resistant fraction (*K*_*0*_ */N*). Panels are grouped by antibiotic administration time (*AB* = 20 or 60), and the right-side labels indicate the network settings used in each block (A–I), where *K* is the number of nearest neighbors initially connected to each node and *β* is the rewiring probability. Values are shown as mean ± SD. **C:** Heatmap showing the mean change in clustering coefficient induced by antibiotic perturbation across simulation replicates (*N* = 100) in Barabási-Albert networks. Within each panel, rows correspond to transmission probability (*p*) and columns correspond to the initial resistant fraction (*K*_*0*_ */N*). Panels are grouped by antibiotic administration time (*AB* = 20 or 60), and the right-side labels indicate the attachment parameter *m*, defined as the number of links added by each new node when joining the network. Values are shown as mean ± SD.

**Figure 6.**
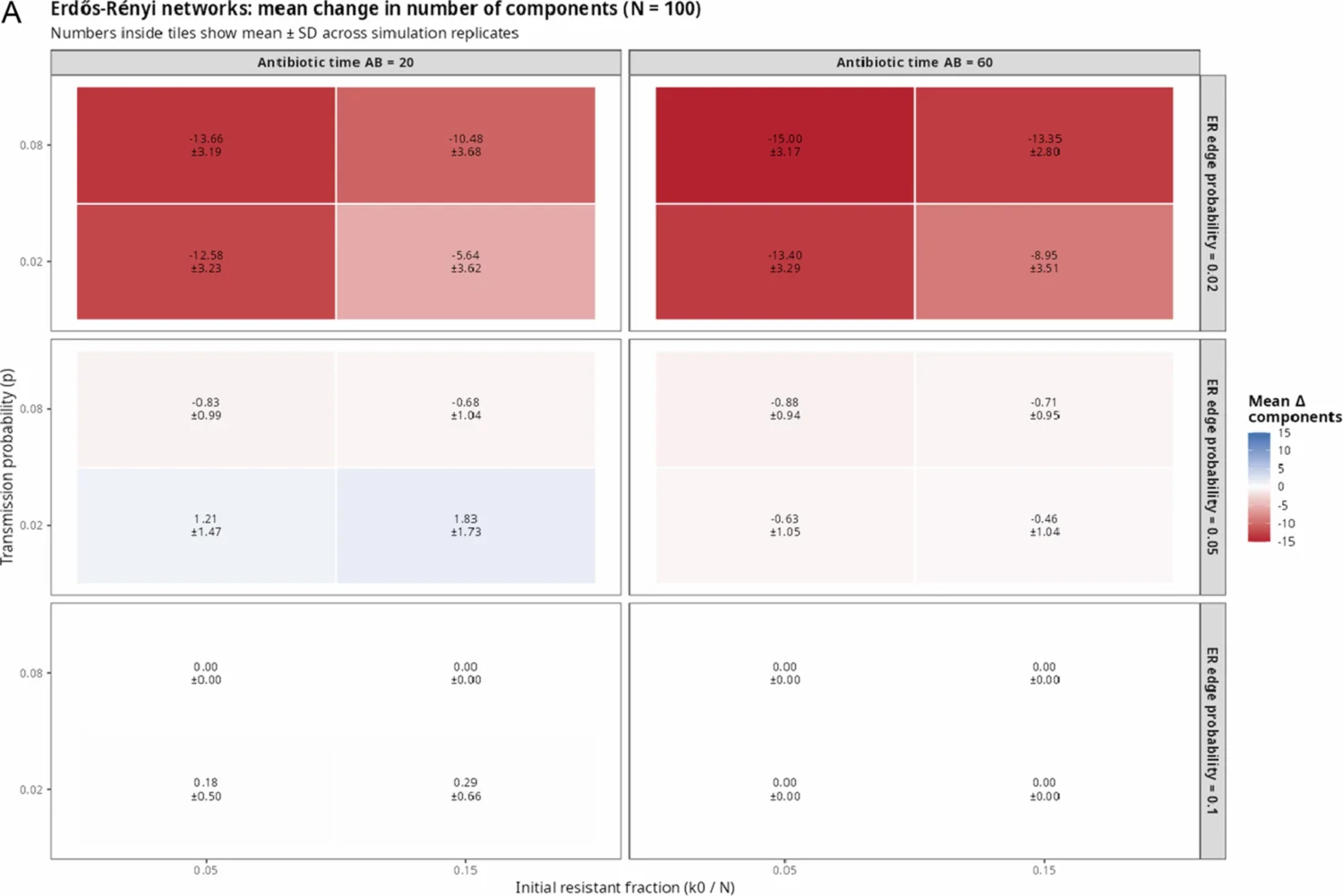
**A**: Heatmap showing the mean change in the number of connected components induced by antibiotic perturbation across simulation replicates (*N* = 100) in Erdős-Rényi networks. Within each panel, rows correspond to transmission probability (*p*) and columns correspond to the initial resistant fraction (*K*_*0*_ */N*). Panels are grouped by antibiotic administration time (*AB* = 20 or 60), and the right-side labels indicate the ER edge probability, that is, the probability that any pair of nodes is connected when the random network is generated. Values are shown as mean ± SD. **B**: Heatmap showing the mean change in the number of connected components induced by antibiotic perturbation across simulation replicates (*N* = 100) in Watts-Strogatz networks. Within each panel, rows correspond to transmission probability (*p*) and columns correspond to the initial resistant fraction (*K*_*0*_ */N*). Panels are grouped by antibiotic administration time (*AB* = 20 or 60), and the right-side labels indicate the network settings used in each block (A–I), where *K* is the number of nearest neighbors initially connected to each node and *β* is the rewiring probability. Values are shown as mean ± SD. **C**: Heatmap showing the mean change in the number of connected components induced by antibiotic perturbation across simulation replicates (*N* = 100) in Barabási-Albert networks. Within each panel, rows correspond to transmission probability (*p*) and columns correspond to the initial resistant fraction (*K*_*0*_ */N*). Panels are grouped by antibiotic administration time (*AB* = 20 or 60), and the right-side labels indicate the attachment parameter *m*, defined as the number of links added by each new node when joining the network. Values are shown as mean ± SD.

The simulations show that antibiotic treatment can substantially alter global network properties, including edge density, clustering, connectivity, and the size of the giant component (as seen in Figures 4A−C, 5A−C, and 6A−C). These structural changes emerge as a direct consequence of which nodes remain in the network after susceptible populations are removed. When resistance remains relatively localized prior to treatment, the elimination of susceptible nodes produces large topological shifts relative to the original network structure. In these regimes, the surviving network contains far fewer interactions, and the connectivity between the remaining resistant populations is strongly reduced.

In biological terms, a reduction in the giant component can be interpreted as a loss of global ecological cohesion among the resistant populations that survive the perturbation. This does not necessarily imply literal species extinction in vivo. In the model, it indicates that the surviving resistant subset is more fragmented, which may correspond to reduced metabolic coupling, loss of shared niches, or increased community vulnerability.

In contrast, when resistance spreads broadly across the network before antibiotic administration, the resulting structural perturbation is much smaller. Because many nodes have already acquired resistance, a larger portion of the original association structure remains intact after treatment, and connectivity among the surviving resistant populations is largely preserved. As a result, global network properties after antibiotic administration remain closer to those of the pre-treatment network.

Notably, the simulations suggest the presence of a transition between these two regimes. Under parameter combinations that limit the spread of resistance, such as low transmission probability or small initial resistant fractions, the antibiotic perturbation produces pronounced topological changes in the network. However, once resistance propagation surpasses certain thresholds, the post-antibiotic network becomes structurally similar to the original network prior to treatment. This behavior reflects the interplay between resistance dynamics and antibiotic-induced percolation: the structural impact of the antibiotic depends critically on how much of the network has already been colonized by resistant populations before treatment occurs.

Together, these results indicate that antibiotic perturbations do not simply reduce the number of populations present in the system but can reorganize the connectivity structure of microbial ecological networks, with the magnitude of this reconfiguration determined by the preceding dynamics of resistance spread.

### Case study: simulating resistance spread on an empirical network from the gut microbiome

**Figure 7.**
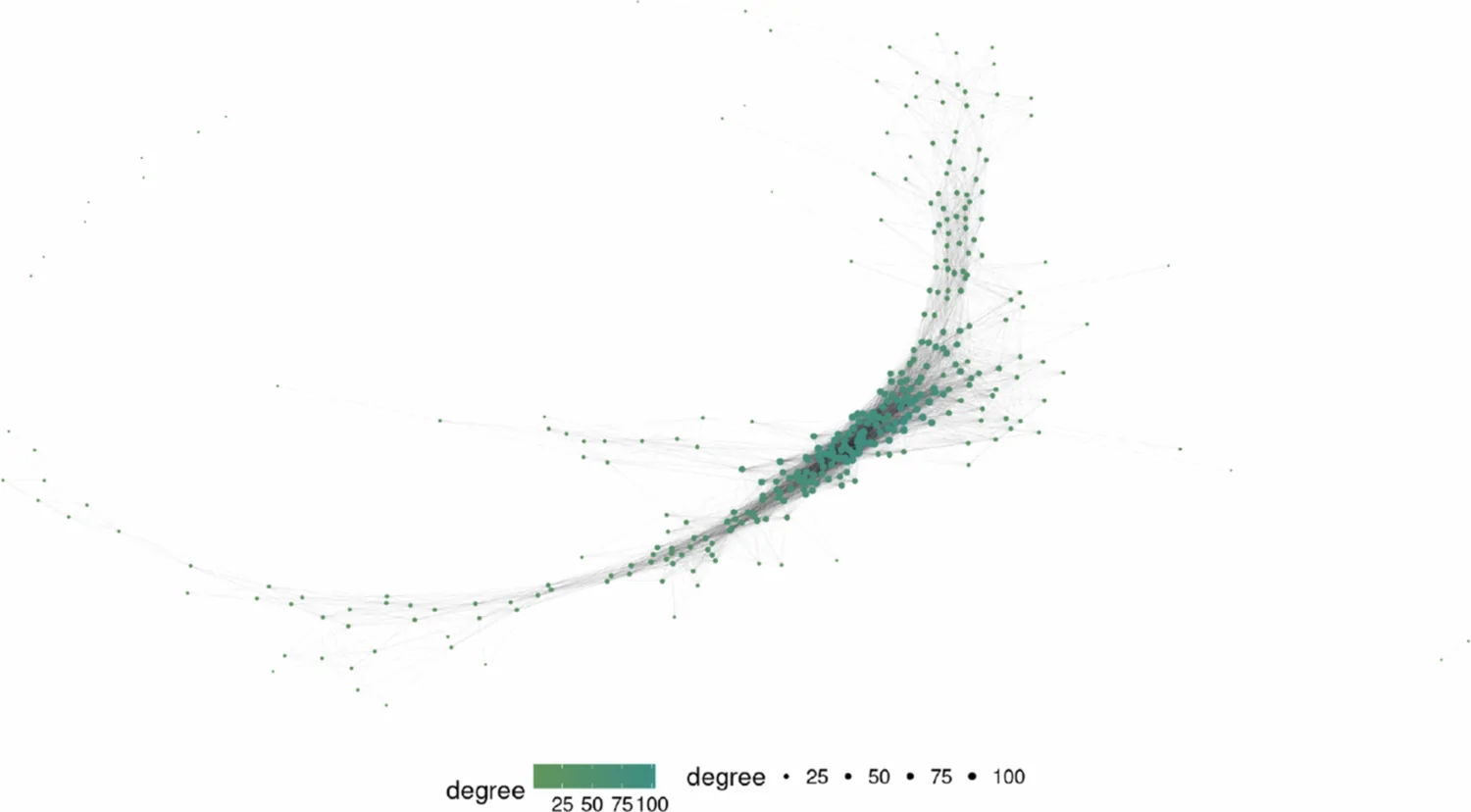
Network visualization of the enterotype network. This network contains 347 nodes and 7,304 edges, organized in 6 components. The nodes represent microbial taxa, while the edges represent ecological proximity (using the Jaccard method as implemented in the {phyloseq} package, using a max distance threshold of 0.3).

To evaluate whether the behaviors observed in synthetic networks also emerge in a biologically grounded example, we simulated resistance dynamics on an empirical microbial association network derived from human gut microbiome data. In contrast to canonical graph models studied in the previous section, the reconstructed empirical network (Figure 7) exhibits a heterogeneous structure that includes uneven degree distributions and several disconnected components under the selected Jaccard threshold. These features are properties of this reconstructed association network and should not be read as a general claim that all human gut microbiomes have the same topology.

**Figure 8.**
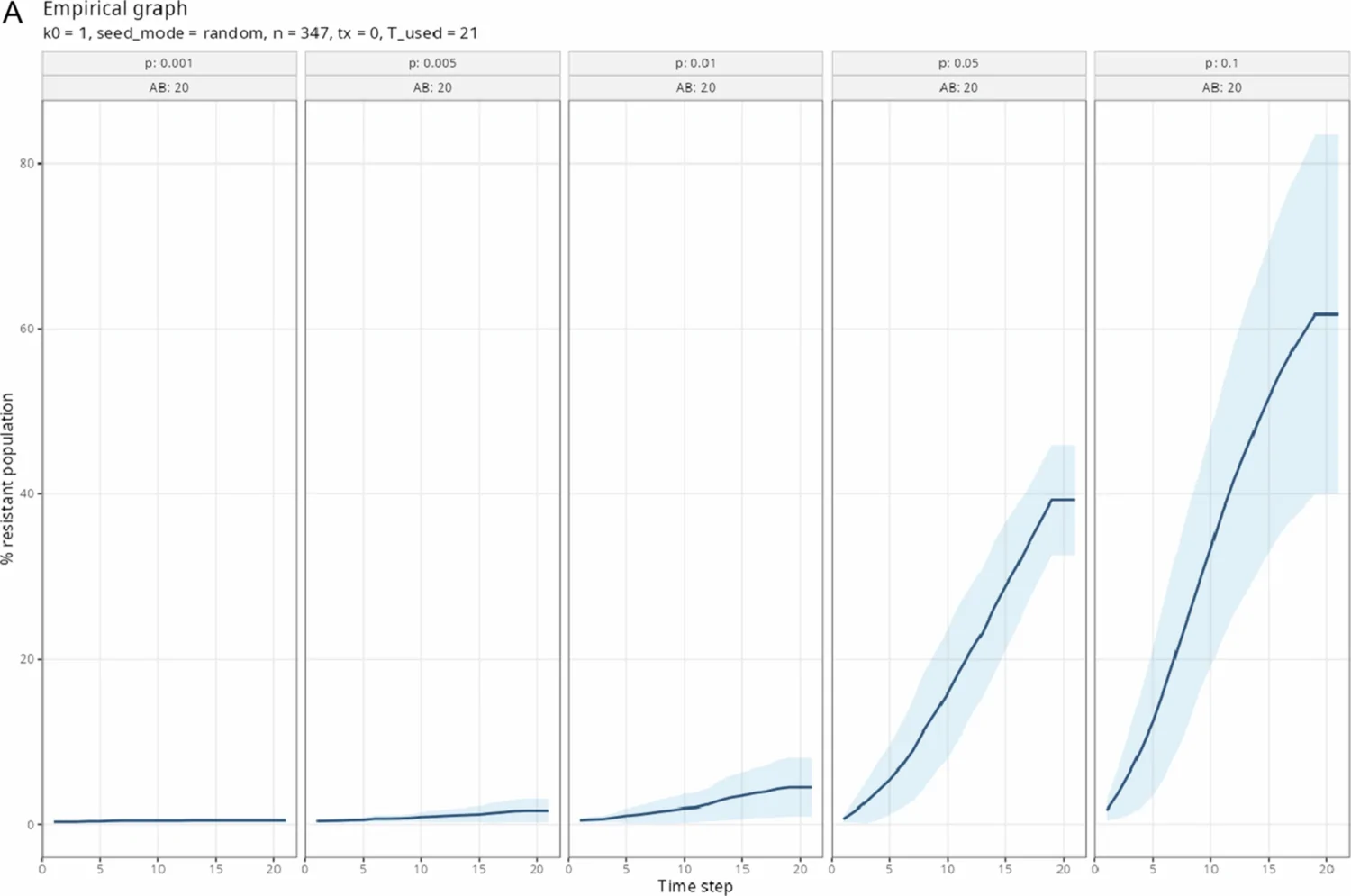
**A:** Time series of resistant population percentage in the empirical microbial association network under random seeding. Resistance dynamics are measured as the percentage of the population that is resistant at a given time step. The shaded area indicates variation across simulation replicates. Panels are organized according to the transmission probability *p*. Initial resistant nodes were selected at random. **B**: Time series of resistant population percentage in the empirical microbial association network under hub seeding. Resistance dynamics are measured as the percentage of the population that is resistant at a given time step. The shaded area indicates variation across simulation replicates. Panels are organized according to the transmission probability *p*. Initial resistant nodes were placed in highly connected nodes. **C**: Time series of resistant population percentage in the empirical microbial association network under dispersed seeding. Resistance dynamics are measured as the percentage of the population that is resistant at a given time step. The shaded area indicates variation across simulation replicates. Panels are organized according to the transmission probability *p*. Initial resistant nodes were distributed far from one another in the network.

The temporal trajectories of resistance spread show a strong dependence on the transmission probability *p* (Figure 8A–C). For very low transmission probabilities (*p* = 0.001 and *p* = 0.005), resistance spreads minimally and remains confined to a small fraction of nodes during the simulated time window. As transmission probability increases, propagation accelerates and reaches progressively larger portions of the network, approaching roughly one third of the nodes under the highest transmission regimes explored.

The empirical simulations also highlight the importance of where resistance is initially introduced in the network. When resistance is seeded at random nodes, propagation proceeds gradually and exhibits considerable variability across simulation replicates (Figure 8A). Seeding resistance in high-degree nodes leads to faster and more consistent expansion, reflecting the large number of ecological associations available for transmission (Figure 8B). In contrast, initializing resistance in dispersed nodes, such that the initial resistant populations are not immediate neighbors, slows the propagation process and leads to more heterogeneous trajectories (Figure 8C). The wider variability observed in these simulations therefore arises primarily from the different seeding strategies explored.

**Figure 9.**
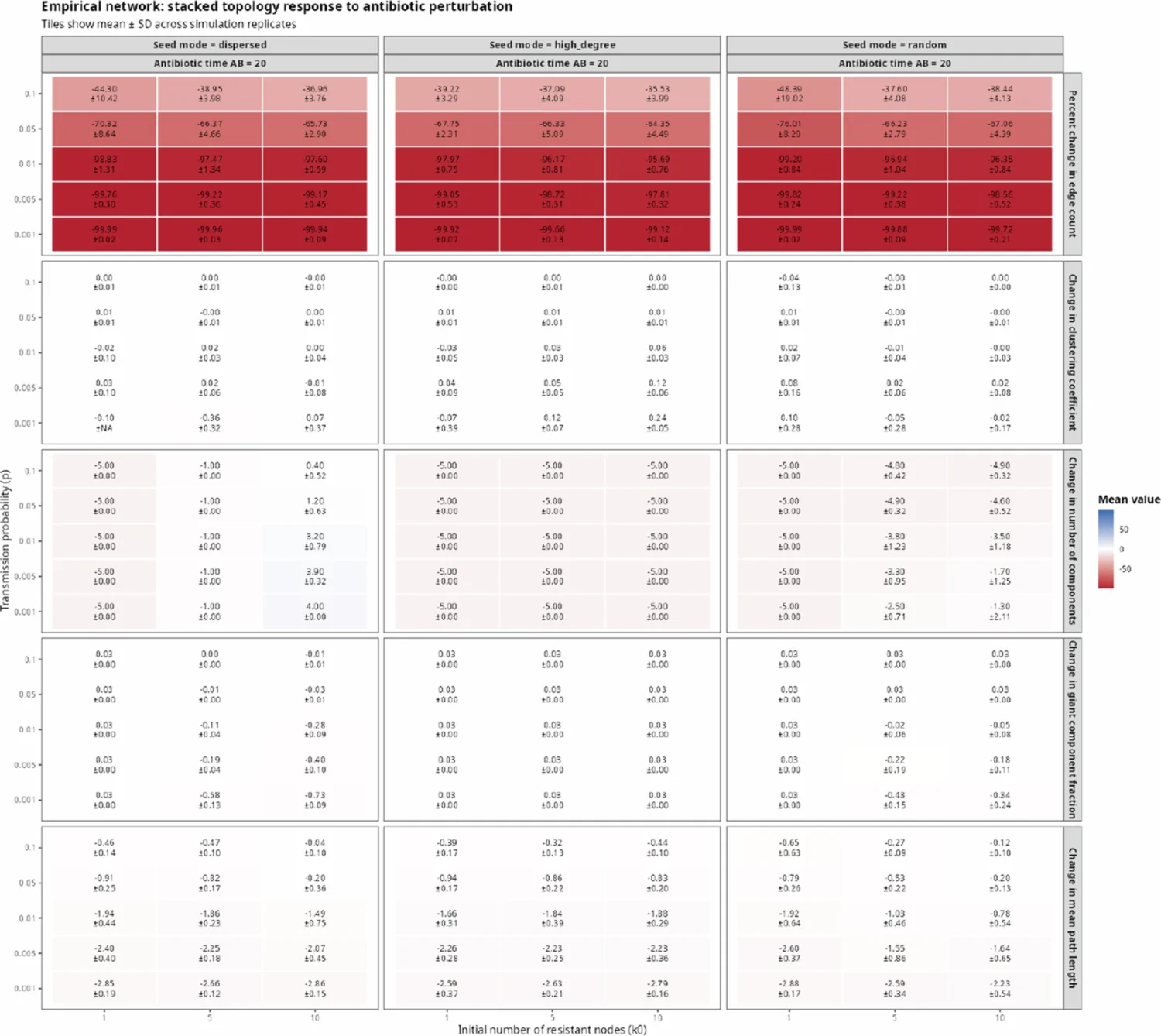
Heatmap showing the mean topological changes induced by antibiotic perturbation across simulation replicates in the empirical microbial interaction network(s). Within each panel, rows correspond to transmission probability (*p)* and columns correspond to the initial number of resistant nodes (*K*_*0*_). Panels are grouped according to the seeding strategy used to initialize resistance: random, hub, or spatially dispersed. The right-side labels indicate the network property change represented in each row block: edge density, clustering coefficient, number of connected components, giant component fraction and mean path length. Values are shown as mean ± SD.

Antibiotic administration produces structural changes in the empirical network that are consistent with the patterns observed in the synthetic models (Figure 9). When resistance remains limited prior to treatment, the removal of susceptible nodes results in large reductions in edge count and connectivity among the remaining populations. Conversely, when resistance spreads more broadly before the antibiotic is applied, a larger portion of interactions between resistant populations persists after treatment, resulting in smaller topological changes.

Together, these results show that the dynamics identified in the synthetic networks also operate in a realistic microbial ecological network. In particular, both the network structure and the initial placement of resistant populations influence the trajectories of resistance spread and the magnitude of the structural reconfiguration induced by antibiotic perturbation.

## Discussion

In this work we developed and analyzed a simple simulation-based network model to study antibiotic resistance in microbial communities. Starting from a first-principles network representation of microbial ecological associations, we modeled bacterial populations or metapopulations as nodes connected by ecological proximity and allowed resistance-associated states to propagate across these links before antibiotic treatment. Across both synthetic network models and an empirical microbial association network, we found that resistance expansion and the structural outcome of antibiotic intervention depend jointly on transmission dynamics, initial resistance conditions, and network topology. These results should be interpreted as qualitative behavior from a minimal model, not as direct validation of a specific molecular mechanism.

A central implication of this framework is that antibiotics can be interpreted not only as selective agents acting on individual microbial populations, but also as perturbations that restructure ecological association networks.^33^ ^,^ ^51^ In the model, antibiotic administration removes all susceptible nodes simultaneously, leaving only resistant populations and the interactions among them. The resulting network therefore represents the ecological structure that survives after treatment. From this perspective, antibiotic therapy effectively performs a large-scale percolation process on the microbial network, and the structure that remains depends on how extensively resistance has spread before the intervention occurs.^52-54^

The present model also omits several environmental and molecular variables known to affect resistance spread. Antibiotic concentration, exposure time, multi-drug exposure, biocides, metals, nutrient availability, temperature, osmolarity, humidity, radiation, coalescent particles, extracellular DNA, and nanoparticles can all influence the availability and movement of resistance genes.^2^ ^,^ ^26^ ^,^ ^55^ ^,^ ^56^ In this study, these factors are collapsed into simplified transmission and perturbation parameters. This simplification is useful for isolating the role of network topology, but it limits direct biological interpretation.

The simulations suggest that the structural impact of antibiotic treatment depends strongly on the dynamical history of resistance spread. When resistance remains limited before treatment, the removal of susceptible populations produces large topological changes in the network, including strong reductions in connectivity and interaction density. In contrast, when resistance spreads widely prior to treatment, the post-antibiotic network remains structurally similar to the original ecological network. This behavior indicates the presence of a transition between regimes in which antibiotic perturbations either strongly disrupt the microbial association structure or leave it largely intact.^57^ ^,^ ^58^ In this sense, the ecological consequences of antibiotic treatment are history dependent: the same intervention can produce very different structural outcomes depending on the prior trajectory of resistance propagation.^59^

The changes in connected components and giant component size may be viewed as indicators of network fragility. In this sense, some microbial association networks may appear robust to small perturbations but fragile when key resistant or susceptible regions are affected. This interpretation is consistent with the broader idea of robust-yet-fragile organization in microbial resistomes and ecological networks, although the present simulations do not test this mechanism directly.^46^ ^,^ ^60^

The behavior observed in the empirical microbial network provides a useful perspective for interpreting recent findings on the long-term effects of antibiotics on the gut microbiome. Several large-scale and longitudinal studies have shown that antibiotic exposure can have persistent consequences for microbial community composition, in some cases lasting for years after treatment.^61^ ^,^ ^62^ At the same time, other studies suggest that microbiomes frequently exposed to antibiotics may recover or reach alternative steady states that are comparatively resilient to subsequent perturbations.^63^ The dynamics observed in our empirical simulations offer a possible network-based interpretation for these seemingly contrasting outcomes. Because resistance-associated states spread across ecological association networks prior to antibiotic exposure, the structural impact of treatment depends strongly on how widely resistance has already propagated within the modeled community. When resistance remains localized, antibiotic administration removes a large portion of the network and induces major structural changes. In contrast, when resistance is already broadly distributed, the remaining network retains much of its association structure and the perturbation becomes comparatively mild. This interpretation should be understood as a conceptual comparison rather than a direct causal validation in human subjects.

More broadly, the empirical simulations reinforce the idea that the effects of antibiotics on the gut microbiome depend strongly on the ecological organization of the microbial community. The gut microbiome is increasingly understood as a complex ecological network whose response to antibiotic exposure can vary substantially across individuals and treatments.^64^ In our model, both the topology of the association network and the location of initial resistant populations influence the trajectory of resistance spread and the structural consequences of antibiotic intervention. This observation is consistent with experimental evidence showing that antibiotics differ in their activity spectra across commensal species and therefore perturb microbial communities in class-specific ways.^65^ Taken together, these results suggest that variability in antibiotic outcomes may partly arise from differences in underlying microbial association networks and in the distribution of resistant taxa within those networks prior to treatment.

External acquisition is another important process that is not explicitly modeled here. Resistant organisms or mobile genetic elements can enter a community from food, water, animals, soil, or other human-associated sources and then interact with resident microbial association networks.^2^ ^,^ ^29^ ^,^ ^55^ In the present model, the different seeding regimes can be interpreted as simplified scenarios for the topological placement of resistance entering a pre-existing microbial association network. In future implementations, external acquisition could be modeled as immigration of resistant nodes, introduction of mobile genetic elements, or time-dependent changes in the initial seeding of resistance.

In this model, resistance acquisition is represented by the emergence of duplicated resistant counterparts, rather than a direct state change of the original node. In the gut microbiome, this can be interpreted as a simplified ecological abstraction of the local spread of resistance, rather than literal cell division, speciation, or gene duplication. However, it is interesting to note that duplicated antibiotic resistance genes have been associated with ongoing selection and horizontal gene transfer in bacterial communities.^21^ In addition, the gut microbiome is recognized as an important reservoir of resistance genes and horizontal transfer events,^18^ and it can undergo restructuring following antibiotic exposure.^62^ Therefore, the topological results should be interpreted within the state-expanded population representation, not as direct measurements of taxonomic diversification or species-level ecological rewiring in a real microbial community.

An advantage of the present approach is that the model can be implemented with relatively simple computational tools. Because the dynamics operate directly on network structures, simulations can be performed using standard graph representations. This simplicity makes the model easy to adapt to different datasets or network types and lowers the barrier for exploring resistance dynamics in ecological networks. In contrast, many existing tools for modeling contagion processes were developed for epidemiological applications and require additional adaptation to represent microbial ecological interactions.^48^

In summary, these results highlight the value of network-based approaches for understanding the ecological consequences of antibiotic resistance. By explicitly representing microbial communities as ecological association networks and modeling resistance-associated states as propagating across modeled ecological links, the framework developed here connects microbial ecology, network dynamics, and antibiotic perturbations within a unified representation. Even under simplified assumptions, the model captures key qualitative features of resistance spread and reveals how the structure of microbial association networks can shape both the dynamics of resistance propagation and the structural outcomes of antibiotic intervention. Together, these findings suggest that incorporating ecological network structure into models of antibiotic resistance may provide a useful perspective for exploring how microbial communities respond to perturbations and how such responses depend on the underlying organization of microbial ecosystems.

## Conclusions and future directions

This study introduces a simple network-based framework to explore how antibiotic resistance propagates through microbial ecological interactions and how antibiotic treatment restructures those networks. Across both synthetic and empirical networks, we show that the spread of resistance and the structural outcome of antibiotic perturbation depend jointly on transmission dynamics, initial resistance conditions, and the topology of the underlying ecological network.

The simulations indicate that antibiotic treatment can be interpreted as a network-level perturbation that removes susceptible populations and reshapes the ecological association structure. Importantly, the magnitude of this restructuring depends on the dynamical history of resistance spread: when resistance remains localized, antibiotic administration produces substantial topological disruption, whereas widespread resistance can preserve much of the original association structure.

Together, these results highlight the importance of considering microbial communities as ecological networks when studying antibiotic resistance. Even minimal mechanistic models can reveal how the association structure of microbial populations shapes resistance dynamics and the ecological consequences of antibiotic interventions.

The present framework was intentionally designed as a minimal proof-of-concept model. Its modular structure allows for straightforward extensions, including explicit compatibility constraints between donor and recipient populations, mobile genetic element host range, plasmid dynamics, fitness costs of resistance, vertical inheritance, abundance-explicit population dynamics, competitive and cooperative interactions among species, and more realistic antibiotic action such as partial efficacy, time-dependent effects, concentration dependence, or multi-drug environments.

Another important opportunity lies in combining dynamical models such as the one proposed here with the growing set of methods used to infer microbial association networks from sequencing data.^31^ ^,^ ^32^ ^,^ ^66^ Numerous statistical approaches exist to reconstruct ecological associations among microbial taxa, yet relatively few empirically derived networks are available for dynamical analysis. Integrating network reconstruction with simulation frameworks could enable researchers to move beyond static descriptions of microbial communities and instead explore how inferred ecological networks respond to perturbations such as antibiotic treatment.

In the longer term, such approaches may contribute to predictive models of microbiome responses to treatment. By reconstructing patient-specific microbial association networks and resistance profiles, it may become possible to simulate potential treatment outcomes before therapy is administered, providing a conceptual pathway toward microbiome-informed digital twins in precision medicine.^67^

## Supplementary Material

supplementary_file_1.zip

supplementary_file_2.zip

## Data Availability

Simulations are reproducible from: https://github.com/guillermodeandajauregui/AntibioticPerturbationNetworks. The full dataset of the simulations used in this paper is available at, doi: 10.5281/zenodo.18992044.

## Addendum A

The simulation framework was implemented in R using {igraph} for network representation and manipulation, and {tidyverse} tools for data handling, parameter sweeps, and result aggregation. For the empirical network analysis, microbiome data were accessed and processed using {phyloseq}.^50^ The codebase was organized into modular components separating graph preparation, resistance dynamics, and simulation orchestration, allowing the same engine to be applied to synthetic networks (ER, WS, BA) and empirical microbial networks.

Each simulation produces a compact set of artifacts designed to facilitate downstream analysis and reproducibility. For every simulation run the following objects are stored: the pre-perturbation network snapshot, the post-perturbation network snapshot, a time series table containing the number of resistant and susceptible nodes at each time step, and simulation metadata describing the parameters used for that run.

Outputs are organized in a directory hierarchy corresponding to parameter sweeps. Each simulation generates a compact file containing the network snapshots and time series results, together with an index table summarizing all simulation runs within the experiment. This design allows efficient storage of large ensembles of simulations while preserving the information necessary to analyze resistance trajectories and pre versus post perturbation network structure.

The following parameters were used in the simulations on synthetic networks:

The parameters *p*, k_0_, and AB should be interpreted as simulation controls. The transmission probability *p* summarizes several biological processes that are not separated in this minimal model, including donor-recipient contact, mobile genetic element compatibility, successful establishment, and the fitness of the resistant state. The antibiotic time AB denotes the update step at which the perturbation is applied, not a calibrated clinical treatment time.

**Table:**

Category	Parameter	Values explored
Network size	n	100, 300, 1000
ER topology	p_edge	0.02, 0.05, 0.10
WS topology	k	4, 8, 12
WS topology	beta	0.01, 0.05, 0.1
BA topology	m	1, 2, 3, 5
Transmission probability	p	0.02, 0.08
Initial resistant fraction	k0_frac	0.05, 0.15
Antibiotic intervention	AB (time of administration)	20, 60
Graph replicates	n_graph	10
Simulation replicates	n_sim	10

Whereas the empirical network, simulations were run with the following parameters.

**Table:**

Category	Parameter	Values explored
Initial resistant nodes	K0	1, 5, 10
Transmission probability	p	0.001, 0.005, 0.01, 0.05, 0.1
Antibiotic intervention	AB (time of administration)	20, 60

## References

[1] Frieri M , Kumar K , Boutin A . Antibiotic resistance. J Infect Public Health. 2017;10(4):369–378. doi: 10.1016/j.jiph.2016.08.007. 27616769

[2] Larsson DGJ , Flach C-F . Antibiotic resistance in the environment. Nat Rev Microbiol. 2022;20(5):257–269. doi: 10.1038/s41579-021-00649-x. PMC8567979 34737424

[3] Zainab SM , Junaid M , Xu N , Malik RN . Antibiotics and antibiotic resistant genes (ARGs) in groundwater: a global review on dissemination, sources, interactions, environmental and human health risks. Water Res. 2020;187:116455. doi: 10.1016/j.watres.2020.116455. 33032106

[4] Pinheiro F . Predicting the evolution of antibiotic resistance. Curr Opin Microbiol. 2024;82:102542. doi: 10.1016/j.mib.2024.102542. 39298866

[5] Sanz-García F , Gil-Gil T , Laborda P , Blanco P , Ochoa-Sánchez L-E , Baquero F , Martínez JL , Hernando-Amado S . Translating eco-evolutionary biology into therapy to tackle antibiotic resistance. Nat Rev Microbiol. 2023;21(10):671–685. doi: 10.1038/s41579-023-00902-5. 37208461

[6] Singer RS , Ward MP , Maldonado G . Can landscape ecology untangle the complexity of antibiotic resistance? Nat Rev Microbiol. 2006;4(12):943–952. doi: 10.1038/nrmicro1553. 17109031

[7] Pons MJ , Ruiz J . Current trends in epidemiology and antimicrobial resistance in intensive care units. J Emerg Crit Care Med. 2019;3:5–5. doi: 10.21037/jeccm.2019.01.05.

[8] Pourmand A , Mazer-Amirshahi M , Jasani G , May L . Emerging trends in antibiotic resistance: implications for emergency Medicine. Am J Emerg Med. 2017;35(8):1172–1176. doi: 10.1016/j.ajem.2017.03.010. 28302376

[9] Blair JMA , Webber MA , Baylay AJ , Ogbolu DO , Piddock LJV . Molecular mechanisms of antibiotic resistance. Nat Rev Microbiol. 2015;13(1):42–51. doi: 10.1038/nrmicro3380. 25435309

[10] Lin J , Nishino K , Roberts MC , Tolmasky M , Aminov RI , Zhang L . Mechanisms of antibiotic resistance. Front Microbiol. 2015;6. doi: 10.3389/fmicb.2015.00034. PMC4318422 25699027

[11] Estrela S , Brown SP . Community interactions and spatial structure shape selection on antibiotic resistant lineages. PLoS Comput Biol. 2018;14(6):e1006179. doi: 10.1371/journal.pcbi.1006179. PMC6013025 29927925

[12] Layeghifard M , Hwang DM , Guttman DS . Disentangling interactions in the microbiome: a network perspective. TIM. 2017;25(3):217–228. doi: 10.1016/j.tim.2016.11.008. PMC7172547 27916383

[13] Nair RR , Andersson DI . Interspecies interaction reduces selection for antibiotic resistance in escherichia coli. Communications Biology. 2023;6(1):331. doi: 10.1038/s42003-023-04716-2. PMC10043022 36973402

[14] Denk-Lobnig M , Wood KB . Antibiotic resistance in bacterial communities. Curr Opin Microbiol. 2023;74:102306. doi: 10.1016/j.mib.2023.102306. PMC10527032 37054512

[15] Murray AK , Zhang L , Yin X , Zhang T , Buckling A , Snape J , Gaze WH . Novel insights into selection for antibiotic resistance in complex microbial communities. mBio. 2018;9(4):e00969-18. doi: 10.1128/mBio.00969-18. PMC6058293 30042197

[16] Diebold PJ , Rhee MW , Shi Q , Trung NV , Umrani F , Ahmed S , Kulkarni V , Deshpande P , Alexander M , Thi Hoa N , et al. Clinically relevant antibiotic resistance genes are linked to a limited set of taxa within gut microbiome worldwide. Nat Commun. 2023;14(1):7366. doi: 10.1038/s41467-023-42998-6. PMC10645880 37963868

[17] Lathakumari RH , Vajravelu LK , Satheesan A , Ravi S , Thulukanam J . Antibiotics and the gut microbiome: understanding the impact on human health. Medicine in Microecology. 2024;20:100106. doi: 10.1016/j.medmic.2024.100106.

[18] McInnes RS , McCallum GE , Lamberte LE , Van Schaik W . Horizontal transfer of antibiotic resistance genes in the human gut microbiome. Curr Opin Microbiol. 2020;53:35–43. doi: 10.1016/j.mib.2020.02.002. 32143027

[19] Xu L , Surathu A , Raplee I , Chockalingam A , Stewart S , Walker L , Sacks L , Patel V , Li Z , Rouse R . The effect of antibiotics on the gut microbiome: a metagenomics analysis of microbial shift and gut antibiotic resistance in antibiotic treated mice. BMC Genomics. 2020;21(1):263. doi: 10.1186/s12864-020-6665-2. PMC7106814 32228448

[20] Hossain AKMZ , Chowdhury AMMA . Understanding the evolution and transmission dynamics of antibiotic resistance genes: a comprehensive review. J Basic Microbiol. 2024;64(10):e2400259. doi: 10.1002/jobm.202400259. 39113256

[21] Maddamsetti R , Yao Y , Wang T , Gao J , Huang VT , Hamrick GS , Son H-I , You L . Duplicated antibiotic resistance genes reveal ongoing selection and horizontal gene transfer in bacteria. Nat Commun. 2024;15(1):1449. doi: 10.1038/s41467-024-45638-9. PMC10873360 38365845

[22] Michaelis C , Grohmann E . Horizontal gene transfer of antibiotic resistance genes in biofilms. Antibiotics. 2023;12(2):328. doi: 10.3390/antibiotics12020328. PMC9952180 36830238

[23] Broaders E , Gahan CGM , Marchesi JR . Mobile genetic elements of the human gastrointestinal tract: potential for spread of antibiotic resistance genes. Gut Microbes. 2013;4(4):271–280. doi: 10.4161/gmic.24627. PMC3744512 23651955

[24] Ghaly TM , Gillings MR . New perspectives on mobile genetic elements: a paradigm shift for managing the antibiotic resistance crisis. Philos Trans R Soc B: Biol Sci. 2022;377(1842):20200462. doi: 10.1098/rstb.2020.0462. PMC8628067 34839710

[25] Partridge SR , Kwong SM , Firth N , Jensen SO . Mobile genetic elements associated with antimicrobial resistance. Clin Microbiol Rev. 2018;31(4):e00088-17. doi: 10.1128/CMR.00088-17. PMC6148190 30068738

[26] Berglund B . Environmental dissemination of antibiotic resistance genes and correlation to anthropogenic contamination with antibiotics. Infect Ecol Epidemiol. 2015;5(1):28564. doi: 10.3402/iee.v5.28564. PMC4565060 26356096

[27] Von Wintersdorff CJH , Penders J , Van Niekerk JM , Mills ND , Majumder S , Van Alphen LB , Savelkoul PHM , Wolffs PFG . Dissemination of antimicrobial resistance in microbial ecosystems through horizontal gene transfer. Front Microbiol. 2016;7. doi: 10.3389/fmicb.2016.00173. PMC4759269 26925045

[28] Wang D , Zhou X , Fu Q , Li Y , Ni B-J , Liu X . Understanding bacterial ecology to combat antibiotic resistance dissemination. Trends Biotechnol. 2025;43(7):1566–1582. doi: 10.1016/j.tibtech.2024.12.011. 39855970

[29] Baquero F , Coque TM , Martínez JL , Aracil-Gisbert S , Lanza VF . Gene transmission in the one health microbiosphere and the channels of antimicrobial resistance. Front Microbiol. 2019;10:2892. doi: 10.3389/fmicb.2019.02892. PMC6927996 31921068

[30] Oña L , Shreekar SK , Kost C . Disentangling microbial interaction networks. TIM. 2025;33(6):619–634. doi: 10.1016/j.tim.2025.01.013. 40044528

[31] Friedman J , Alm EJ . Inferring correlation networks from genomic survey data. PLoS Comput Biol. 2012;8(9):e1002687. doi: 10.1371/journal.pcbi.1002687. PMC3447976 23028285

[32] Kurtz ZD , Müller CL , Miraldi ER , Littman DR , Blaser MJ , Bonneau RA . Sparse and compositionally robust inference of microbial ecological networks. PLoS Comput Biol. 2015;11(5):e1004226. doi: 10.1371/journal.pcbi.1004226. PMC4423992 25950956

[33] Röttjers L , Faust K . From hairballs to hypotheses–biological insights from microbial networks. FEMS Microbiol Rev. 2018;42(6):761–780. doi: 10.1093/femsre/fuy030. PMC6199531 30085090

[34] Danon L , Ford AP , House T , Jewell CP , Keeling MJ , Roberts GO , Ross JV , Vernon MC . Networks and the epidemiology of infectious disease. Interdiscip Perspect Infect Dis. 2011;2011:1–28. doi: 10.1155/2011/284909. PMC3062985 21437001

[35] Masuda N , Holme P . Introduction to temporal network epidemiology. In EN Masuda , P Holme (Eds.), Temporal Network Epidemiology. 2017. pp. 1–16 Springer Singapore. doi: 10.1007/978-981-10-5287-3_1.

[36] Trevisin C , Mari L , Gatto M , Colizza V , Rinaldo A . Epidemiological indices with multiple circulating pathogen strains. Infect Dis Model. 2025;10(3):802–812. doi: 10.1016/j.idm.2025.03.006. PMC11985023 40213021

[37] Pastor-Satorras R , Vespignani A . Epidemic spreading in scale-free networks. PhRvL. 2001;86(14):3200–3203. doi: 10.1103/PhysRevLett.86.3200. 11290142

[38] Pastor-Satorras R , Castellano C , Van Mieghem P , Vespignani A . Epidemic processes in complex networks. Rev Mod Phys. 2015;87(3):925–979. doi: 10.1103/RevModPhys.87.925.

[39] Newman M , Barabási A-L , Watts DJ . The Structure and Dynamics of Networks. 2011. Princeton University Press. doi: 10.1515/9781400841356.

[40] Martínez JL , Baquero F . Emergence and spread of antibiotic resistance: setting a parameter space. Ups J Med Sci. 2014;119(2):68–77. doi: 10.3109/03009734.2014.901444. PMC4034563 24678768

[41] Song LY , D’Souza S , Lam K , Kang TM , Yeh P , Miller JH . Exploring synergy between classic mutagens and antibiotics to examine mechanisms of synergy and antibiotic action. Antimicrob Agents Chemother. 2016;60(3):1515–1520. doi: 10.1128/AAC.02485-15. PMC4775987 26711761

[42] Baquero F , Coque TM . Multilevel population genetics in antibiotic resistance. FEMS Microbiol Rev. 2011;35(5):705–706. doi: 10.1111/j.1574-6976.2011.00293.x. 21714793

[43] Witzany C , Rolff J , Regoes RR , Igler C . The pharmacokinetic–pharmacodynamic modelling framework as a tool to predict drug resistance evolution: this article is part of the microbial evolution collection. Microbiology. 2023;169(7). doi: 10.1099/mic.0.001368. PMC10433423 37522891

[44] Chowdhury AR , Venkateswaran J , Damani OP . Hybrid agent-based and system dynamics modeling of anti-bacterial resistance in community. In 2025 Winter Simulation Conference (WSC). 2025. pp. 1083–1094. doi: 10.1109/WSC68292.2025.11338910.

[45] Eklöf A , Jacob U , Kopp J , Bosch J , Castro‐Urgal R , Chacoff NP , Dalsgaard B , De Sassi C , Galetti M , Guimarães PR , et al. The dimensionality of ecological networks. Ecol Lett. 2013;16(5):577–583. doi: 10.1111/ele.12081. 23438174

[46] Montoya JM , Pimm SL , Solé RV . Ecological networks and their fragility. Natur. 2006;442(7100):259–264. doi: 10.1038/nature04927. 16855581

[47] Landi P , Minoarivelo HO , Brännström [ , Hui C , Dieckmann U . Complexity and stability of ecological networks: a review of the theory. Popul Ecol. 2018;60(4):319–345. doi: 10.1007/s10144-018-0628-3.

[48] Miller J , Ting T . EoN (Epidemics on Networks): a fast, flexible python package for simulation, analytic approximation, and analysis of epidemics on networks. J Open Source Softw. 2019;4(44):1731. doi: 10.21105/joss.01731.

[49] Arumugam M , Raes J , Pelletier E , Le Paslier D , Yamada T , Mende DR , Fernandes GR , Tap J , Bruls T , Batto J-M , et al. MetaHIT Consortium (additional members) Enterotypes of the human gut microbiome. Natur. 2011;473(7346):174–180. doi: 10.1038/nature09944. PMC3728647 21508958

[50] McMurdie PJ , Holmes S . Phyloseq: an R package for reproducible interactive analysis and graphics of microbiome census data. PLoS One. 2013;8(4):e61217. doi: 10.1371/journal.pone.0061217. PMC3632530 23630581

[51] Coyte KZ , Schluter J , Foster KR . The ecology of the microbiome: networks, competition, and stability. Sci. 2015;350(6261):663–666. doi: 10.1126/science.aad2602. 26542567

[52] Albert R , Jeong H , Barabási A-L . Error and attack tolerance of complex networks. Natur. 2000;406(6794):378–382. doi: 10.1038/35019019. 10935628

[53] Callaway DS , Newman MEJ , Strogatz SH , Watts DJ . Network robustness and fragility: percolation on random graphs. PhRvL. 2000;85(25):5468–5471. doi: 10.1103/PhysRevLett.85.5468. 11136023

[54] Li M , Liu R-R , Lü L , Hu M-B , Xu S , Zhang Y-C . Percolation on complex networks: theory and application. Phys Rep. 2021;907:1–68. doi: 10.1016/j.physrep.2020.12.003.

[55] Baquero F , Coque TM , Guerra-Pinto N , Galán JC , Jiménez-Lalana D , Tamames J , Pedrós-Alió C . The influence of coalescent microbiotic particles from water and soil on the evolution and spread of antimicrobial resistance. Front environ sci. 2022;10:824963. doi: 10.3389/fenvs.2022.824963.

[56] Chowdhury NN , Hicks E , Wiesner MR . Investigating and modeling the regulation of extracellular antibiotic resistance gene bioavailability by naturally occurring nanoparticles. Environ Sci Technol. 2022;56(21):15044–15053. doi: 10.1021/acs.est.2c02878. PMC9979080 35853206

[57] Dakos V , Matthews B , Hendry AP , Levine J , Loeuille N , Norberg J , Nosil P , Scheffer M , De Meester L . Ecosystem tipping points in an evolving world. Nature Ecology & Evolution. 2019;3(3):355–362. doi: 10.1038/s41559-019-0797-2. 30778190

[58] Scheffer M , Carpenter S , Foley JA , Folke C , Walker B . Catastrophic shifts in ecosystems. Natur. 2001;413(6856):591–596. doi: 10.1038/35098000. 11595939

[59] Hao Y , Wang X , Liu J , Wu M , Du J , Xue K , Song X , Cui X , Zhao T , Wang Y . Generalized mechanism model for ecosystem hysteresis. Adv Sci. 2026;13:e09008. doi: 10.1002/advs.202509008. PMC13042370 41532545

[60] Fernández-de-Bobadilla MD , Pérez-Cobas AE , Andremont A , Martínez JL , Baquero F , Lanza VF , Coque TM . The antimicrobial gut resistome of the wayampi reveals a shared background of antibiotic- and metal-resistance genes with industrialized populations, underscoring the robust-yet-fragile architecture of human gut microbiomes. Microbiome. 2026;14(1):93. doi: 10.1186/s40168-026-02345-5. PMC13020191 41803907

[61] Baldanzi G , Larsson A , Sayols-Baixeras S , Dekkers KF , Hammar U , Nguyen D , Graells T , Ahmad S , Gazolla Volpiano C , Meric G , et al. Antibiotic use and gut microbiome composition links from individual-level prescription data of 14,979 individuals. Nat Med. 2026;32:1351–1361. doi: 10.1038/s41591-026-04284-y. PMC13099378 41814006

[62] Palleja A , Mikkelsen KH , Forslund SK , Kashani A , Allin KH , Nielsen T , Hansen TH , Liang S , Feng Q , Zhang C , et al. Recovery of gut microbiota of healthy adults following antibiotic exposure. Nat Microbiol. 2018;3(11):1255–1265. doi: 10.1038/s41564-018-0257-9. 30349083

[63] Bich VTN , Le NG , Barnett D , Chan J , Van Best N , Tien TD , Anh NTH , Hoang TH , Van Doorn HR , Wertheim HFL , et al. Moderate and transient impact of antibiotic use on the gut microbiota in a rural Vietnamese cohort. Sci Rep. 2022;12(1):20189. doi: 10.1038/s41598-022-24488-9. PMC9691687 36424459

[64] Fishbein SRS , Mahmud B , Dantas G . Antibiotic perturbations to the gut microbiome. Nat Rev Microbiol. 2023;21(12):772–788. doi: 10.1038/s41579-023-00933-y. PMC12087466 37491458

[65] Maier L , Goemans CV , Wirbel J , Kuhn M , Eberl C , Pruteanu M , Müller P , Garcia-Santamarina S , Cacace E , Zhang B , et al. Unravelling the collateral damage of antibiotics on gut bacteria. Natur. 2021;599(7883):120–124. doi: 10.1038/s41586-021-03986-2. PMC7612847 34646011

[66] Liu C , Cui Y , Li X , Yao M . Microeco: an R package for data mining in microbial community ecology. FEMS Microbiol Ecol. 2021;97(2):fiaa255. doi: 10.1093/femsec/fiaa255. 33332530

[67] De Domenico M , Allegri L , Caldarelli G , d’Andrea V , Di Camillo B , Rocha LM , Rozum J , Sbarbati R , Zambelli F . Challenges and opportunities for digital twins in precision Medicine from a complex systems perspective. Npj Digital Medicine. 2025;8(1):37. doi: 10.1038/s41746-024-01402-3. PMC11742446 39825012

